# PROTOCOL: Breakfast consumption, anthropometry, and nutrition‐related outcomes in adolescents from low‐ and middle‐income countries: Protocol for a systematic review and meta‐analysis

**DOI:** 10.1002/cl2.1415

**Published:** 2024-05-28

**Authors:** Jordie A. J. Fischer, Jonathan Thomas, Kesso Gabrielle van Zutphen‐Küffer, Despo Ierodiakonou, Klaus Kraemer, Vanessa Garcia‐Larsen

**Affiliations:** ^1^ Sight and Life Basel Switzerland; ^2^ School of Medicine, Texas A&M Bryan Texas USA; ^3^ Department of Human Nutrition & Health Wageningen University & Research Wageningen The Netherlands; ^4^ Department of Primary Care and Population Health University of Nicosia Medical School Nicosia Cyprus; ^5^ Program in Human Nutrition, Department of International Health Johns Hopkins University Baltimore Maryland USA

## Abstract

Objectives

This is the protocol for a Campbell systematic review. The objectives are as follows: The aim of this systematic review is to examine the scientific evidence available from low‐ and middle‐income countries on the association of breakfast consumption habits and anthropometry/adiposity‐ and nutrition‐related outcomes in adolescents aged 10–19 years old.

## 1 BACKGROUND

### 1.1 The problem, condition, or issue

There are approximately 1.8 billion adolescents aged 10–19 years worldwide, totaling one‐sixth of the world's population (UNICEF, 2012). Nearly 90% of adolescents live in low‐ and middle‐income countries (LMICs), among which many face a nutrition crisis (United Nations, 2017). The triple burden of malnutrition, characterized by the co‐existence of undernutrition (wasting and/or stunting), micronutrient deficiencies, and overnutrition (overweight and obesity), exists among adolescents in every country (United Nations, 2017). It is acknowledged that healthy dietary patterns play critical roles in total energy intake, weight management, and the burden of non‐communicable diseases. Breakfast eating is a proposed dietary factor influencing body weight, energy intake, and nutritional status (Ricotti et al., 2021).

Breakfast consumption may be of pivotal nutritional value during adolescence, a life stage marked by critical growth and development. Yet, adolescents often sacrifice this mealtime. Recent evidence on adolescent girls’ dietary patterns in LMICs revealed that 40% reported skipping breakfast (defined as anything other than daily consumption), with this behavior being more common among adolescent girls aged 15–19 years (49%) compared to those aged 10–14 years (40%) (Keats et al., 2018). Regional differences have been identified, with a greater frequency of breakfast‐skippers residing in Africa (47%) and East Asia and the Pacific (41%), compared to Latin America and Caribbean adolescents (17%) (Keats et al., 2018). A systematic review of 33 countries (of which 23 were high‐income nations) reported that 10–30% of adolescents skip breakfast, and is more common in girls (Keats et al., 2018; Monzani et al., 2019). This high prevalence is concerning as the nutritional consequences of missing breakfast have been described in several observational and some intervention studies and can have significant implications for the health and well‐being of adolescents in LMICs (Onyiriuka et al., 2013; Ropitasari et al., 2020; Zahedi et al., 2022).

### 1.2 The intervention

Definitions and dietary compositions of breakfast vary across countries and cultures. In addition, the concept of breakfast skipping differs across multiple studies. Breakfast skipping can occur for various reasons, with lack of time, appetite, body weight control, and household food insecurity being the most commonly cited (Esquius et al., 2021; Feye et al., 2023; Hearst et al., 2016; Onyiriuka et al., 2013; Wijtzes et al., 2015).

### 1.3 How the intervention might work

Regardless of the reason, this behavior has been associated with detrimental effects in adolescents, as breakfast skipping is a known risk factor for anemia (Abalkhail & Shawky, 2002; Ropitasari et al., 2020) and odds of anxiety, depression, and psychological distress (Zahedi et al., 2022). Moreover, breakfast skipping has been found to be associated with unhealthy lifestyles in adolescents, particularly with smoking (Kapantais et al., 2011) and poorer dietary quality (O'Neil et al., 2015). On the contrary, regularly consuming breakfast has been associated with increased physical activity, (Corder et al., 2014) better overall diet quality (Chitra & Reddy, 2007; Giménez‐Legarre et al., 2020), and reduced risk of being overweight (Ahadi et al., 2015; Ardeshirlarijani et al., 2019; Arora et al., 2012; Deshmukh‐Taskar et al., 2010). There is contradictory scientific evidence regarding whether breakfast skipping results in a compensatory increase in energy intake later in the day, but a 2019 meta‐analysis of randomized controlled trials of adults in high‐income countries revealed a modest difference (mean difference 259.79 kcal/day) (Sievert et al., 2019). Breakfast meals containing carbohydrates and fiber (whole grains, fruits) may support a consumption pattern associated with lower BMI, potentially due to increased satiety and decreased subsequent energy intake through metabolic changes of diminished postprandial glycemic and insulinemic response, increased insulin sensitivity, and reduced between‐meal hypoglycemia (Cho et al., 2003; Liljeberg et al., 1999; Nestler et al., 1988; Pereira & Ludwig, 2001). Moreover, complex carbohydrates may affect the release or activity of digestive hormones, including cholecystokinin (CCK), that may further influence satiety (Bourdon et al., 1999; Pereira & Ludwig, 2001). A trial of healthy subjects and individuals with type 2 diabetes showed breakfast skipping to acutely disrupt circadian rhythms in both groups by adversely affecting clock and clock‐controlled gene expression. This disruption was correlated with increased postprandial glycemic response in both groups, showing the potential role of skipping breakfast in increased BMI and diabetes (Jakubowicz et al., 2017). There is also evidence of the association of breakfast consumption with improved cognitive function and academic performance (Adolphus et al., 2013). Adolescents may be particularly sensitive to the nutritional effects of breakfast on brain activity and associated cognitive outcomes, as children have higher brain glucose metabolism compared to adults (Adolphus et al., 2016). Additionally, the extended fasting period due to heightened sleep demands during adolescence may further deplete glycogen stores overnight. With glucose being a primary energy source for the brain, a well‐balanced breakfast may provide the glucose needed to fuel brain activity (Adolphus et al., 2016; Ferrer‐Cascales et al., 2018).

### 1.4 Why it is important to do this review

Although trends in adolescents’ cardiometabolic health and body weight status in response to breakfast habits have been documented in individual countries, with the majority conducted in high‐income countries and systematic reviews, little comparable information exists on global trends specific to LMICs where malnutrition is prevalent. To our knowledge, a review has yet to be done on the breakfast‐skipping behaviors of adolescents in LMICs and their effect on anthropometric, adiposity, and nutrition outcomes. Evidence from LMICs, where the detrimental effect of insufficient diets remains a major public health challenge, is urgently needed to inform public policy and to establish nutritional priorities and interventions (e.g., school breakfast programs, public awareness campaigns, fortification of staple breakfast foods) to support countries where the burden of the problem is greatest. The results from this review will shed light on the anthropometric and nutritional consequences of breakfast skipping and will inform policymakers and public health experts to develop targeted interventions to promote healthy, regular breakfast consumption.

## 2 OBJECTIVES

The aim of this systematic review is to examine the scientific evidence available from LMICs on the association of breakfast skipping and consumption habits and anthropometry/adiposity‐ and nutrition‐related outcomes in adolescents aged 10–19 years old.

## 3 METHODS

### 3.1 Criteria for considering studies for this review

#### 3.1.1 Types of studies

Experimental and observational studies will be included. No year limits will be applied to searches.

#### 3.1.2 Types of participants

The definition of an adolescent varies among different organizations. The World Health Organization (WHO) defines an adolescent as anyone between 10 and 19 years, inclusive (World Health Organization). In our study, we will adhere to this definition and include studies with participants aged 10–19 years from LMICs as categorized by the World Bank (The World Bank). All adolescents from this age group will be included regardless of health status and gender. Studies with a participant sample that partially includes the age group of interest will be included if the mean age of the sample is between 10 and 19 years, inclusive. Data extracted from this subset of studies will be considered to represent the 10–19 age group unless disaggregated adolescent data is available and will be included instead.

#### 3.1.3 Types of interventions

Studies will be included if they have a breakfast‐skipping group (intervention/exposed group) and a breakfast‐consuming group (control/nonexposed group). We will accept studies assessing breakfast skipping as a binary variable (yes/no) or based on the frequency (e.g., days with/without breakfast per week). Given the various definitions possible for breakfast skipping, the behavior was defined according to the definition applied by the study authors. Studies will be excluded if they only assessed lunch, dinner, or snack as defined by study authors, skipped other meals besides breakfast, did not include a control group (breakfast consumption group, as defined by study authors), or if co‐interventions are not balanced across both breakfast habit groups (e.g., iron supplementation given to breakfast consuming group, but not to skipping group).

#### 3.1.4 Types of outcome measures

Briefly describe the types of outcome measures that will be included and excluded.

##### Primary outcomes

Considering indicators to measure adolescent health, the following primary outcomes will be extracted.

###### Anthropometry/adiposity outcomes

Body mass index (BMI) (defined as weight in kilograms (kg) divided by height in meters squared), BMI‐for‐age (thinness, *z*‐scores), and weight status (defined categorically as underweight, normal weight, pre‐obesity/overweight, and obese according to the Centers for Disease Control and Prevention) (Centers for Disease Control and Prevention, 2023).

###### Nutritional outcomes

Frequency (%) of anemia (defined as Hemoglobin (Hb) < 115 g/L (11.5 g/dL or 7.14 mmol/L) for boys and girls 10–11 years old, Hb <120 g/L (12.0 g/dL or 7.4 mmol/L) for boys 12–14 years old and girls 12–19 years old, and Hb <130 g/L (13.0 g/dL or 7.7 mmol/L) for boys 15–19 years old, as per WHO guidelines) (World Health Organization, 2011) and Hb serum levels (measured in any units).

##### Secondary outcomes

The following secondary outcomes will be extracted. The nutritional‐specific biomarkers below were chosen as outcomes of interest due to the common deficiency of these micronutrients among adolescents (Akseer et al., 2017; Kumar et al., 2018).

###### Anthropometry/adiposity outcomes

Waist circumference, waist‐to‐height ratio, waist‐to‐hip ratio, height‐for‐age (stunting, *z*‐scores), skinfold thickness, hip circumference, MUAC (10–14 years), and bioelectric impedance.

###### Nutritional outcomes

Frequency (%) of iron deficiency (defined as serum ferritin <15 µg/L, as per WHO guidelines), (World Health Organization, 2020) and iron‐deficiency anemia (relevant sub‐population Hb cutoff + serum ferritin <15 µg/L), iron levels as assessed by ferritin or soluble transferrin receptor (sTfR), vitamin A levels as serum/plasma retinol or retinol‐binding protein concentrations, iodine levels as urinary iodine, zinc levels as plasma zinc, and calcium and vitamin D levels as 25(OH)D, parathyroid hormone or total calcium in serum/plasma or urine.

#### 3.1.5 Duration of follow‐up

We will include studies with any duration of follow‐up; for longitudinal studies with repeated outcome measurements, we will include the timepoint with the lowest attrition and/or estimates from mixed models for longitudinal analysis.

#### 3.1.6 Types of settings

We will include studies from LMIC settings, as defined by the World Bank in 2023 (The World Bank). The World Bank annually assigns countries to four income levels: low‐income, lower‐middle income, upper‐middle income, and high‐income. This review will include countries classified as low‐income, lower‐middle‐income, and upper‐middle‐income.

### 3.2 Search methods for identification of studies

Guided by the Campbell Searching for Studies Guide, (Kugley et al., 2017) we will attempt to identify and retrieve both published and unpublished studies through a comprehensive search that includes electronic databases, sources of gray literature, registries, relevant reference lists, and websites.

#### 3.2.1 Electronic searches

The following databases will be searched: MEDLINE PubMed, Ovid EMBASE, CINAHL, CENTRAL, and Web of Science without language limits. The search strategy will be tailored to each database based on a combination of three concepts: breakfast habits, adolescents, and LMICs. We chose not to include primary or secondary outcomes in our search strategy in line with recommendations due to search sensitivity, as the outcome of interest may not always be mentioned in abstracts or subject headings (Duyx et al., 2019; Frandsen et al., 2020; Tsujimoto et al., 2022). A sample comprehensive search strategy for EMBASE can be found in Table S1 (Supporting Information 1).

#### 3.2.2 Searching other resources

We will search for gray literature in the International Standard Randomized Controlled Trial Number Registry, ProQuest Dissertations and Theses, ClinicalTrials.gov and internet searches using Google's advanced search tool. We will also examine the reference lists of included studies to identify further relevant work and relevant systematic review reference lists. All studies identified through electronic searches or methods detailed here will be retrieved from the Texas A&M Library.

### 3.3 Data collection and analysis

#### 3.3.1 Description of methods used in primary research

The systematic review will be carried out according to the Preferred Reporting System for Systematic Reviews and Meta‐Analysis (PRISMA) Guidelines (Page et al., 2021).

#### 3.3.2 Selection of studies

Studies identified from the search strategy will be imported into Covidence, an online systematic review management software. Duplicates will be removed, and two independent reviewers (J.T. and J.A.J.F.) will screen studies in two phases according to the inclusion criteria detailed above: first by title and abstract and second by full‐text. Any discrepancies between the two reviewers will be resolved by a third reviewer (V.G.L.).

#### 3.3.3 Data extraction and management

Data from eligible studies will be extracted independently by two authors (J.T. and J.A.J.F.) using a data extraction form, which will be piloted with the two authors to ensure clarity and comprehensiveness (Supporting Information 2). Data to be extracted from all eligible studies include author and year of publication, study country and study year and/or period, study design, sample size, participant characteristics (age, sex, source population), details on exposure/intervention and outcomes including method and timing of assessment, and breakfast consumption/skipping definitions used. Any discrepancies between the two authors will be resolved by a third author (V.G.L.). When information regarding any of the studies is unclear, or data is unavailable, we will contact the study authors for further details.

#### 3.3.4 Assessment of risk of bias in included studies

Bias will be assessed at both the study and outcome levels. The Cochrane risk‐of‐bias tool for randomized trials (RoB 2) (Sterne et al., 2019) will be used to examine potential sources of bias across multiple domains in RCTs. The Risk of Bias in Non‐randomized Studies of Interventions (ROBINS‐I) tool (Sterne et al., 2016) will assess the risk of bias in non‐randomized studies. The NIH Quality Assessment Tool for Observational Cohort and Cross‐Sectional Studies (National Institutes of Health, 2014) will assess the risk of bias for observational studies. The risk of bias for each study will be assessed by two independent reviewers (J.T. and J.A.J.F.) utilizing the appropriate screening tool, and a third independent reviewer (V.G.L.) will resolve any conflicts.

#### 3.3.5 Measures of treatment effect

Observational studies reporting odds ratios (ORs) will be synthesized separately from those reporting Risk Ratios (RRs) or Hazard Ratios (HRs). For continuous outcomes, if mean (SD) is reported in different groups instead of beta‐coefficient, then we will estimate mean difference and standard error (SE), where possible, and pool those with studies reporting *β*‐coefficient and SE or 95% confidence interval (95% CI). We will harmonize effect size measures when necessary for meta‐analyses of continuous outcomes (e.g., standardized mean difference) (Haidich, 2010) and consult with statistical support when considering combining ORs and RRs/HRs.

#### 3.3.6 Unit of analysis issues

A unit of analysis issue may arise if there is no dichotomous breakfast‐consuming and ‐skipping group data, but instead multiple groups across a spectrum of breakfast‐consuming frequency. The group with the most limited breakfast consumption pattern will be termed the “infrequent breakfast consumer” group. The reference group will be the one with the highest frequency of breakfast consumption and termed “regular breakfast consumers.” All other groups (other than most and least consumption of breakfast) will be combined to create a single pair‐wise comparison as the “irregular breakfast consumers.” Studies reporting breakfast skipping as a binary variable will be pooled separately.

#### 3.3.7 Criteria for determination of independent findings

To ensure that the effects of an individual exposure/intervention are only counted once, in the case of multiple participant cohorts in one study, each cohort will be treated as a separate study contributing a single effect size estimate to the meta‐analysis, as long as the groups do not share a control group.

#### 3.3.8 Dealing with missing data

If data is not readily calculable or available, study authors will be contacted to retrieve the necessary information. If data cannot be obtained, the study will be excluded from the meta‐analysis but retained for the narrative synthesis. Further, missing data will also be assessed as part of risk of bias.

#### 3.3.9 Assessment of heterogeneity

Study heterogeneity will be estimated using the I^2^ index in conjunction with visual inspections of forest plots and considering methodological differences between studies.

#### 3.3.10 Assessment of reporting biases

Publication bias will be assessed visually using funnel plots, with funnel plot asymmetry suggesting publication bias, and statistically with Egger's test (Egger et al., 1997) for analyses that contain more than 10 studies.

#### 3.3.11 Data synthesis

Trials will be synthesized separately from observational studies. Results of the individual studies will be pooled using the generic inverse variance method. A random effect meta‐analysis will be performed, thereby weighting studies by their sample size. A meta‐analysis will be conducted for each outcome measure. Standardized mean differences (SMD) with 95% CI will be calculated for continuous outcomes, and OR with 95% CIs for dichotomous outcomes. We will present effect estimates with 95% CI, and a heterogeneity analysis, with the threshold for statistical significance will be set at *p* = 0.05. Pooled estimates for different observational study designs will be presented in forest plots separately (prospective vs. retrospective/cross‐sectional studies), together with the overall pooled estimate. When meta‐analysis is not feasible, data will be synthesized following the Synthesis Without Meta‐Analysis (SWiM) guidelines (Campbell et al., 2020).

#### 3.3.12 Subgroup analysis and investigation of heterogeneity

We will investigate possible sources of heterogeneity by performing subgroup analysis on the overall risk of bias and age at outcome.

#### 3.3.13 Sensitivity analysis

Sensitivity analysis will be carried out by excluding studies with two or more domains identified as having a high or unclear risk of bias from the meta‐analysis or studies including subjects outside the age range of 10–19 years and re‐running the results to ensure robustness. Sensitivity analyses will depend on data availability.

#### 3.3.14 Treatment of qualitative research

We do not plan to include qualitative research.

#### 3.3.15 Summary of findings and assessment of the certainty of the evidence

The certainty of the evidence will be graded using the Grading of Recommendations Assessment, Development and Evaluation (GRADE) approach (Guyatt et al., 2008). Based on the GRADE evaluation, the quality of the body of evidence will be graded as “high,” “moderate,” “low,” or “very low” by outcome. Several GRADE domains (e.g., risk of bias, inconsistency, imprecision, indirectness, or publication bias) will be considered, and based on these, evidence may be downgraded or upgraded with transparent reasoning. A summary of findings table will be created using GRADEpro GDT. This information will inform the confidence in the effect estimates.

## CONTRIBUTIONS OF AUTHORS

All authors have read and approved the above protocol.

JAJF and JT drafted the content of the manuscript.

KGvZ, DI, KK, and VGL provided their expert opinion on our content.

JAJF and VGL provided methodological expertise on systematic reviews.

DI and VGL will offer the statistical expertise necessary to execute the analysis.

JT will provide information retrieval expertise.

## DECLARATIONS OF INTEREST

The authors have no conflict of interest to declare.

## PRELIMINARY TIMEFRAME

Training and pilot testing on the inclusion criteria = 2 weeks

Searches for eligible studies = 1 week

Screening the results from the literature search = 2 weeks

Training and pilot testing the study coding procedure = 1 week

Extraction of data from eligible research reports = 3 weeks

Statistical analysis = 3 weeks

Preparation of the final review report = 8 weeks

## PLANS FOR UPDATING THIS REVIEW

The authors do not intend to update the review upon publication.

## SOURCES OF SUPPORT

### Internal sources

- The authors have no sources of support to declare. New Source of support, Other

The authors have no sources of support to declare.

### External sources

- The authors have no sources of support to declare.

## REGISTRATION AND PROTOCOL

The review protocol was registered with the International Prospective Register of Systematic Reviews (PROSPERO: CRD42023442910).

## DATA, CODE, AND OTHER MATERIALS

N/A.

## Supporting information

Supporting information.

Supporting information.
