# Supplementary material for: PROTOCOL: Breakfast consumption, anthropometry, and nutrition‐related outcomes in adolescents from low‐ and middle‐income countries: Protocol for a systematic review and meta‐analysis
Source: Campbell Syst Rev. 2024 May 28;20(2):e1415. doi: 10.1002/cl2.1415 (PMC11131355; doi:10.1002/cl2.1415)
Supplement: Supplementary file 1 — Supporting information. [file CL2-20-e1415-s002.docx]

# Search strategy

Please provide more details about whether hand searches will be conducted or whether the review will search conference proceedings, government documents, or dissertations. Specify if website searches or search engines will be utilized and explain the process. The protocol needs to clarify if the systematic review will apply or not language limits. Additional resources that could be useful for this review would be CINAHL, PsycINFO, and trial registers such as Clinical trials.gov and the WHO ICTRP portal. You could check Cochrane´s list of LMIC databases.

Author’s Response (AR): We have included these important clarifications within the manuscript text: “The following databases will be searched: MEDLINE PubMed, Ovid EMBASE, CINAHL, CENTRAL, and Web of Science without language limits.

We will search for grey literature in the International Standard Randomized Controlled Trial Number Registry, ProQuest Dissertations and Theses, and ClinicalTrials.gov.”

Comments about the MEDLINE PubMed Search Strategy (Supplementary Table 1): I suggest removing line 2(breakfast skipping) of this search as it could lead to missing relevant studies.

Breakfast skipping is a concept that can appear in different ways. For example, you are missing in that line: miss, intermittent, omission, morning fasting. Therefore, I suggest removing this line. If you retrieve too many records after removing line 2, you could always consider focusing on a type of publication study such as randomized controlled trials (you can apply methodological filters) or you could apply a date limit, for example, the last 20 years (justifying a change in the breakfast behavior during the last years) Other considerations: Please, mention the use of the Campbell guideline for the methodology section. The protocol states that the reference lists of included studies will be checked. Please, consider checking also the reference list of systematic reviews that could be identified.

AR: This feedback on the specificity of our search strategy is very helpful. Each electronic database search strategy was developed in close collaboration with an Informationist at the Welch Medical Library at Johns Hopkins University. After consulting with this expert further and trialling the suggested search strategy changes, we have decided not to adjust the current search strategy. Each search strategy was tailored for the specific database, with line 2 of the EMBASE search strategy being: breakfast*.mp.

Thank you for mentioning the Campbell guideline within the methodology section. The manuscript now appropriately states: “Guided by the Campbell Searching for Studies Guide, [Kugley et al., 2016] we will attempt to identify and retrieve both published and unpublished studies through a comprehensive search, including electronic databases, sources of grey literature, registries, and relevant study and review reference lists and websites.”

To your final point, we will search the reference lists of relevant systematic reviews and have included this in the text: “We will also examine the reference lists of included studies to identify further relevant work and relevant systematic review reference lists.”

There is no mention of Campbell’s Searching for Studies Guide (Kugley et al., 2016)

AR: Before we did not include any introductory text under the “Search methods for identification of studies”. We now open this section with, “Guided by the Campbell Searching for Studies Guide,[Kugley et al., 2016] we will attempt to identify and retrieve both published and unpublished studies through a comprehensive search including electronic databases, sources of grey literature, registries and relevant study and review reference lists and websites.”

Will the authors search for conference proceedings, government documents or dissertations separately? Will the authors conduct a hand search of journals relevant to the topic? Did the authors consult the list of websites in the Campbell Searching for Studies Guide (Kugley et al., 2016)? Do the authors describe their plan for using web search engines (e.g. Google, Yahoo Search, Bing, Duck Duck Go, etc.) to locate web-based material? Do the authors describe how they will use Google Scholar or other freely available scholarly search engines? Do the authors state the platform in which the database will be searched? Will non-English studies be included?

AR: We have grouped the above questions together. Please see the following excerpts of text from the protocol to respond to each question:

“Guided by the Campbell Searching for Studies Guide [Kugley et al., 2016], we will attempt to identify and retrieve both published and unpublished studies through a comprehensive search that includes electronic databases, sources of grey literature, registries, relevant reference lists, and websites.”

“The following databases will be searched: MEDLINE PubMed, Ovid EMBASE, CINAHL, CENTRAL, and Web of Science without language limits. The search strategy will be tailored to each database based on a combination of 3 concepts: breakfast habits, adolescents, and LMICs.”

“We will search for grey literature in the International Standard Randomized Controlled Trial Number Registry, ProQuest Dissertations and Theses, ClinicalTrials.gov and internet searches using Google's advanced search tool. We will also examine the reference lists of included studies to identify further relevant work and relevant systematic review reference lists. All studies identified through electronic searches or methods detailed here will be retrieved from the Texas A&M Library.”

# Methods

There were a few methods-related topics that would benefit from additional detail. There was a discussion of different ways that breakfast skipping may be articulated in each study (e.g., binary vs counts). How could this impact the intervention/effect and the appropriate effect size? More specifics on the specific forms and types of effect size computation would be beneficial. This is especially true given the variety of designs that are being extracted and possibly combined. Without more detail, it is challenging to establish how easily the effect sizes can be combined. The specifics regarding the combination of pairwise comparisons from studies that may have multiple groups are unclear. More detail on this aspect would help understand the process for combination and whether this is appropriate for combination. Finally, the specifics of moderator analyses were short and underdeveloped. Some rationale for the choices and theory of why the two moderators would be of interest would have helped. Furthermore, are there important moderators missing due to the variety of interventions of studies that will be combined? Would the LMIC definition for levels be a possible moderator?

AR: The authors are grateful for these thoughtful comments on the protocol's methodology. In regard to the appropriate effect size, we have restated the use of SMD and ORs in-text:

“Standardized mean differences (SMD) with 95% confidence intervals (95% CI) will be calculated for continuous outcomes and odds ratios (OR) with 95% CIs for dichotomous outcomes. We will present effect estimates with 95%CI, and a heterogeneity analysis, with the threshold for statistical significance will be set at p = 0.05.”

Our previous Unit of Analysis section may have been unclear; therefore, we have clarified the text to read: “A unit of analysis issue may arise if there is no dichotomous breakfast-consuming and -skipping group data, but instead multiple groups across a spectrum of breakfast-consuming frequency. The group with the most limited breakfast consumption pattern will be termed the “breakfast skipping group". The reference group will be the one with the highest frequency of breakfast consumption. All other groups (other than most and least consumption of breakfast) will be combined to create a single pair-wise comparison.”

Thank you for your question regarding moderator analyses, including the appropriateness of subgroup analyses based on economy classifications (LIC as compared to MIC). We see the need to conduct this review in two overlooked populations in regard to data collection and policy: adolescents and LMIC. Therefore, we do not see the relevance of conducting further LMIC subgroup analyses at this time, but regional differences may be of interest if a sufficient number of studies are identified.

Specific comments related to items on the MECCIR document:

C21: Details on the type of meta-analytic model appropriate for the data extracted was not defined. More details about the analytic plan would help the protocol.

AR: Random effects statistical models using the generic inverse variance method will be used throughout unless a compelling case arises for fixed effect analysis. A random effects model assumes that a treatment effect in each study is randomly selected from a normal distribution and that it varies from study to study (Borenstein, M., Hedges, L. V., Higgins, J. P. T., & Rothstein, H. R. (2009). Introduction to meta-analysis. Wiley).

C66: What would happen to the data if a study had multiple intervention groups/cohorts if the same control group was used for comparison?

AR: As guided by the Cochrane Handbook (Chapters 9 and 16), while there are several possible approaches to including a study with multiple intervention groups in a meta-analysis it must be avoided to simply enter several comparisons into the meta-analysis when these have one or more intervention groups in common as this results in double-counting the participants in the shared group, thus creating a unit-of-analysis error due to the unaddressed correlation between the estimated intervention effects from multiple comparisons. Therefore, Cochrane recommends combining groups to create a single pair-wise comparison, which is what we aim to do.

Our previous Unit of Analysis section may have been unclear; therefore, we have clarified the text to read: “A unit of analysis issue may arise if there is no dichotomous breakfast-consuming and -skipping group data, but instead multiple groups across a spectrum of breakfast-consuming frequency. The group with the most limited breakfast consumption pattern will be termed the “breakfast skipping group". The reference group will be the one with the highest frequency of breakfast consumption. All other groups (other than most and least consumption of breakfast) will be combined to create a single pair-wise comparison.”

# Reviewer 1:

The paragraph on how the intervention might work is fragmented and does not take into account the impacts of breakfast consumption (or skipping) on health and nutrition outcomes, cognitive performance, academic achievement, well-being, and risk factors for NCDs.

All these components you listed are highly important associations documented in the academic literature of breakfast skipping/consumption among adolescents. This paragraph discusses cognitive performance and academic achievement” “There is also evidence of the association of breakfast consumption with improved cognitive function and academic performance.[52] Adolescents may be particularly sensitive to the nutritional effects of breakfast on brain activity and associated cognitive outcomes, as children have higher brain glucose metabolism compared to adults.[7]”. Commenting on risk factors for NCDs, we state: “A trial of healthy subjects and individuals with type 2 diabetes showed breakfast skipping to acutely disrupt circadian rhythms in both groups by adversely affecting clock and clock-controlled gene expression. This disruption was correlated with increased postprandial glycemic response in both groups, showing the potential role of skipping breakfast in increased BMI and diabetes.[54]”. To introduce this habit's impacts on health and well-being, we state: “Moreover, breakfast skipping has been found to be associated with unhealthy lifestyles in adolescents, particularly with smoking[27] and poorer dietary quality.[13] On the contrary, regularly consuming breakfast has been associated with increased physical activity,[51] better overall diet quality[49, 50] and reduced risk of being overweight.[48, 47, 45, 46]. The nutritional impacts of this dietary behaviour are interwoven throughout the paragraph, but we make specific mentions.

when we state, “breakfast skipping is a known risk factor for anemia [43, 44]”.

It is unclear why one of the search strategy concepts includes breakfast skipping rather than breakfast consumption/habit.

AR: Each electronic database search strategy was developed in close collaboration with an Informationist at the Welch Medical Library at Johns Hopkins University. Each strategy was tailored for the specific database, with line 2 of the EMBASE search strategy being “breakfast*.mp,” which keeps the search general to breakfast habits to include both skipping and consumption behaviours.

Searching the gray literature is unclear.

AR: We agree details of our grey literature search were unclear. Please see the revised text:

“We will search for grey literature in the International Standard Randomized Controlled Trial Number Registry, ProQuest Dissertations and Theses, ClinicalTrials.gov and internet searches using Google's advanced search tool. We will also examine the reference lists of included studies to identify further relevant work and relevant systematic review reference lists.”

For missing data, it is unclear what course of action will be taken if the contacted authors do not respond.

AR: We agree that the next steps following an author's non-response were unclear. We now state: “If data is not readily calculable or available, study authors will be contacted to retrieve the necessary information. If data cannot be obtained, the study will be excluded from the meta-analysis but retained for the narrative synthesis.”

Recent evidence on adolescent girls’ dietary patterns in LMICs revealed that 40% reported skipping breakfast,4 with an age-related decline…’4’ does not make sense here

AR: The ‘4’ was an error and has been removed.

## Reviewer 2:

“Breakfast consumption is of pivotal nutritional value during adolescence…”

This claim seems to be a bit of an exaggeration, at least without a reference. If anything, it seems as though this is what the review is trying to investigate. Essentially, if we know that breakfast is of pivotal importance during adolescence, then what would be the purpose of the SR/MA? I did review the Keats et al. paper, and could not find the source for this claim, but it’s possible I missed it.

AR: We agree that this controversial claim is the very statement this review is seeking to investigate. Therefore, we feel it is more appropriate to state: “Breakfast consumption *may* be of pivotal nutritional value during adolescence, a life stage marked by critical growth and development.”

- “…40% reported skipping breakfast, 4…” I assume this sentence is citing the Keats paper, but the in-text citation seems incorrect.

AR: The ‘4’ was an error and has been removed.

- “with an age-related decline” This reads as though as they get older, there is a decline in breakfast skipping (i.e. breakfast consumption increases), but the reference after says the opposite. I’d suggest rewording to be clearer.

AR: This has now been adjusted for improved readability. “Recent evidence on adolescent girls’ dietary patterns in LMICs revealed that 40% reported skipping breakfast (defined as anything other than daily consumption), with this behavior being more common among adolescent girls aged 15-19 years (49%) compared to those aged 10–14 years (40%).”

- “with this behaviour [being] more common in girls” Add in “being”.

AR: We have added “being” into the text.

- “Breakfast skipping is thought to cause a compensatory increase in energy intake later in the day, including more snacking between meals.[22]”

This has been studied before and a 2019 meta-analysis has suggested the opposite (though, the difference was somewhat modest; 79-441 kcal/day)1. Further, when we consider diets such as Intermittent Fasting (which often is characterized by skipping breakfast, and perhaps other meals), research tends to show the same thing (i.e. skipping meals = less caloric intake)2…I will say I don’t believe the question is fully answered yet, but research seems to support that skippers consume less calories. Also, while it has been suggested to increase calories at other meals, it doesn’t seem to add enough to offset the lack of breakfast calories, so if that is the point this is trying to make I think it’s somewhat of a half-truth.

▪ 1. Sievert K, Hussain SM, Page MJ, Wang Y, Hughes HJ, Malek M, et al. Effect of breakfast on weight and energy intake: Systematic review and meta-analysis of randomised controlled trials. BMJ. 2019 Jan 30;364.

2. Welton S, Minty R, O’Driscoll T, Willms H, Poirier D, Madden S, et al. Intermittent fasting and weight loss: Systematic review. Can Fam Physician [Internet]. 2020 Feb 1 [cited 2024 Feb 12];66(2):117. Available from: /pmc/articles/PMC7021351/

AR: Thank you for addressing the inconclusive nature of this research question. In line with the most updated evidence, we have changed this sentence to reflect the contradictory evidence and include the 2019 meta-analysis findings.

“There is contradictory scientific evidence regarding whether breakfast skipping results in a compensatory increase in energy intake later in the day, but a 2019 meta-analysis of randomized controlled trials of adults in high-income countries revealed a modest difference (mean difference 259.79 kcal/day).”

- “The breakfast meal is commonly high in carbohydrates and fiber (whole grains, fruits)…”
Is this true for children/teens as well? Particularly, the whole grains and fruits part? It’s my understanding that breakfast often includes sugary ready-to-eat cereals, which are often lower in fiber than alternatives, and most are not whole grain. The one studied cited here is NHANES 2003 (USA) and uses data from 1988-1994 which is quite dated at this point. I found this study (Alexy U, Wicher M, Kersting M. Breakfast trends in children and adolescents: frequency and quality. Public Health Nutrition. 2010;13(11):1795-1802. doi:10.1017/S1368980010000091) which is also quite a bit dated (1986-2007), but it found that most teens were having “bread meals”, followed by ready-to-eat-cereals. So yes, probably high in carbs, but I don’t think these references suggest teens are consuming fruits and whole grains. I’d suggest removing, and/or finding newer references for these claims.

- “a consumption pattern associated with lower BMI potentially due to increased satiety and decreased subsequent energy intake through metabolic changes of diminished postprandial glycemic and insulinemic response, increased insulin sensitivity, and reduced between-meal hypoglycemia”
I think this is a bit of a stretch. If anything, we know that fat and protein tend to satiate more than carbohydrates. This is especially true when we consider “simple” carbohydrates, so I’m not sure this connection with high carbohydrate = more satiety is relevant. Again, I think these references may still be true today, but it would be advisable to find newer ones with more recent research.

AR: Thank you for noting the lack of data on the current breakfast consumption trends of children and adolescents in LMICs, due to the increasing impacts of the nutrition transition of the diets of children and adolescents. I agree, some of the references stated (4) are outdated. On account of this gap in the literature, we have revised the text to state: “Breakfast meals containing carbohydrates and fiber (whole grains, fruits) may support a consumption pattern associated with lower BMI, potentially due to increased satiety and decreased subsequent energy intake through metabolic changes of diminished postprandial glycemic and insulinemic response, increased insulin sensitivity, and reduced between-meal hypoglycemia.[12,11, 10, 9]”

- “Adolescents may be particularly sensitive to the nutritional effects of breakfast on brain activity and associated cognitive outcomes, as children have higher brain glucose metabolism compared to adults”
This sentence seems very vague. What are the nutritional effects of breakfast? As compared to the nutritional effects of any other meal/snack?

We recognize the sensitivity to nutritional effects may not be unique to breakfast alone and agree this is essential to comment as we interpret the review’s findings. Other meals, such as lunch and dinner, also contribute to overall nutrient intake and can impact cognitive function and energy levels. Yet, breakfast holds significance because it breaks the overnight fasting period. For adolescents, who often have early school start times, breakfast becomes particularly important as it replenishes energy stores and provides essential nutrients after a long fasting period during sleep. Research has shown that consuming breakfast, especially a well-balanced one, can positively affect cognitive function in adolescents.

Peña-Jorquera H, Campos-Núñez V, Sadarangani KP, Ferrari G, Jorquera-Aguilera C, Cristi-Montero C. Breakfast: A Crucial Meal for Adolescents' Cognitive Performance According to Their Nutritional Status. The Cogni-Action Project. Nutrients. 2021 Apr 16;13(4):1320. doi: 10.3390/nu13041320. PMID: 33923639; PMCID: PMC8073030.

- “Definitions and dietary compositions of breakfast vary across countries and cultures. In addition, the concept of breakfast skipping differs across multiple studies. Breakfast skipping can occur for various reasons, with lack of time, appetite and body weight control being the most commonly cited.”

- Good! The definition of breakfast is hotly contested to the point that there is no one singly accepted definition. However, I think it’s also worth mentioning that teens living in lower socioeconomic households tend to skip breakfast more often, and that “skipping” breakfast may not always be a conscious choice (and may be a result of lack of food), especially in LMIC.

AR: We agree that mentioning food insecurity, especially in the context of LMIC settings, is important. We have included additional text and two study references to support this finding. “Breakfast skipping can occur for various reasons, with lack of time, appetite, body weight control, and household food insecurity being the most commonly cited.”

- “Given the various definitions possible for breakfast skipping, the behaviour was defined according to the definition applied by the study authors”
Given the heterogeneity with which breakfast is defined, I would strongly recommend including each study’s definition of “breakfast”, if you already have not planned on doing so, for purposes of possible re-analysis/stratification.

AR: We agree that this information on each study’s definition of breakfast consumption/skipping is essential to capture. “Data to be extracted from all eligible studies include author and year of publication, study country and study year and/or period, study design, sample size, participant characteristics (age, sex, source population), details on exposure/intervention and outcomes including method and timing of assessment, and breakfast consumption/skipping definitions used.”

- “Nutritional Outcomes”

o Anemia is tightly associated with energy intake, so if breakfast skippers are consuming less calories, then that is likely why anemia might occur more often…However, I suspect that would also be true if other meals were skipped as well, and wonder why skipping breakfast might lead to anemia, whereas skipping other meals wouldn’t? If that isn’t the case (i.e. skipping any meal leads to anemia more often), then to me, this seems like less of a breakfast issue and more of a energy intake/expenditure issue. Essentially, is there something inherently unique about the breakfast meal that skipping it could cause a unique increase in risk to developing anemia? And by that logic, if one consumes breakfast, is it necessarily the breakfast that reduces their risk, or is it the additional calories? I would recommend you consider isocaloric diets where possible, due to the mediating nature by which energy intake has on anemia risk. In fact, I think all of the outcomes are going to be associated with adequate energy intake (e.g. BMI, waist circumference, etc.), so it is certainly something to consider. I think it would also be useful to state what type of anemia (e.g. folate, B12, iron) is likely to develop, to better pinpoint where the likely differences are

AR: We agree that a low energy intake can contribute to a low intake of micronutrients such as iron. Therefore, energy intake or expenditure may be a confounding factor in the relationship between breakfast habits and anemia. We will ensure that we unpack this and other potential confounding factors within the review’s discussion. We agree that only selecting studies with isocaloric comparison groups, but on account of an informal preliminary review of the literature, this data is very uncommonly reported and thus will be discussed and noted as a methodological limitation if relevant. Such factors will be taken into consideration during the risk of bias assessments. Confounding factors are a threat in observational studies; to study the effects of skipping breakfast on anthropometric and nutrition outcomes, relevant confounders will be accounted for where adjusted analyses are available. We agree stating the type of anemia reported in each study’s population is necessary data. This data will be collected as a secondary outcome, as noted under the Secondary Nutritional Outcomes section.
